# Medium-Chain Triglyceride Emulsion with Phytocannabinoids and Monolaurin Improves Growth and Survival in Suckling Piglets

**DOI:** 10.3390/ani15192881

**Published:** 2025-10-01

**Authors:** Adisak Kongkeaw, Wandee Tartrakoon, Sonthaya Numthuam, Tossaporn Incharoen, Noraphat Hwanhlem, Juan J. Loor, Rangsun Charoensook

**Affiliations:** 1 Department of Agricultural Science, Division of Animal Science and Feed Technology, Faculty of Agriculture, Natural Resources and Environment, Naresuan University, Phitsanulok 65000, Thailand; adisakk64@nu.ac.th (A.K.); sonthayan@nu.ac.th (S.N.); tossaporni@nu.ac.th (T.I.); noraphath@nu.ac.th (N.H.); 2 Department of Animal Sciences, Division of Nutritional Sciences, University of Illinois, Urbana, IL 61801, USA; jloor@illinois.edu

**Keywords:** medium-chain triglycerides, phytocannabinoids, monolaurin, neonatal piglets, antibiotic alternatives

## Abstract

**Simple Summary:**

Pre-weaning mortality is a persistent challenge in pig production, particularly among low-birth-weight piglets from hyper-prolific sows. To support early growth and survival, this study evaluated a medium-chain triglyceride emulsion (MCTE) and its derivatives supplemented with hemp-derived phytocannabinoids (MCTE-P) or with both phytocannabinoids and monolaurin (MCTE-PM). Compared with antibiotic prophylaxis, emulsion-supplemented piglets showed faster growth, greater colostrum and milk intake, and reduced mortality. MCTE-PM shows clearer benefit in reduced diarrhea-related deaths, improved blood parameters, and alleviated aggressive suckling behaviors such as teat competition and facial lesions. These findings suggest that MCT-based emulsions, particularly MCTE-PM, represent promising nutritional alternatives to antibiotics for improving neonatal piglet health and welfare.

**Abstract:**

This study aimed to evaluate the effects of medium-chain triglyceride (MCT) emulsions enriched with hemp-derived phytocannabinoids, with or without monolaurin, on neonatal piglet growth, health, and behavior. Trial 1 used an augmented factorial design with 75 sows and 1063 piglets to compare a baseline MCT emulsion (MCTE) with a phytocannabinoid-supplemented emulsion (MCTE-P) at low or high doses against toltrazuril control. All MCT emulsions improved key performance indicators such as weight gain and survival rates compared to the control group. In particular, live-born piglets at 24 h in the MCTE-P groups showed significantly greater body weight gain and colostrum intake compared with controls (*p* < 0.05). While overall pre-weaning mortality rates were similar across groups, the incidence of diarrhea- and starvation-related deaths was significantly lower in MCTE-P piglets (*p* < 0.05). Based on these results, Trial 2 involved 36 sows and 509 piglets assigned to three groups: low-dose MCTE-P (the optimal regimen from Trial 1), low-dose MCTE-P supplemented with monolaurin (MCTE-PM), and a toltrazuril control. Both MCTE-P and MCTE-PM improved average daily gain at weaning relative to the control group. MCTE-PM showed the lowest pre-weaning mortality (14.3%) and diarrhea-related deaths (0.86%), compared with 29.4% and 10.4% in controls, respectively (*p* < 0.05). Hematological analyses indicated that eosinophil percentages were lowest in the MCTE-PM group (*p* < 0.05), while serum total protein and globulin concentrations remained elevated in emulsion-treated piglets (*p* < 0.001). Behavioral assessments of 108 low-birth-weight piglets showed prolonged latency to first suckling in emulsion-treated groups, while teat competition and facial lesion scores, reflecting aggressive interactions, were reduced compared with controls. Overall, these findings demonstrate that MCT emulsions supplemented with phytocannabinoids and monolaurin improved growth and survival in neonatal piglets, especially those of low to medium birth weight, and highlight their potential as nutraceutical alternatives to antibiotic prophylaxis in swine production.

## 1. Introduction

Pre-weaning mortality remains a critical challenge in modern swine production, arising from multiple causes such as crushing, inadequate milk intake, diarrhea, and gastrointestinal infections [1]. This issue has become more pronounced with the rapid genetic selection for hyper-prolific sows, producing litters of more than 16 piglets and over 30 weaned piglets per sow each year. While these advances have increased productivity, they have also led to a higher incidence of low-birth-weight piglets (<1.20 kg) [2], which are particularly vulnerable due to limited energy reserves, reduced thermoregulation, and slower access to the udder [3]. Consequently, pre-weaning mortality rates can exceed 20% in hyper-prolific breeds, compared with only 10–12% in conventional lines [4]. This high mortality is strongly associated with the limited viability of low-birth-weight piglets, highlighting the critical role of early nutrition. Since colostrum and sow’s milk are the exclusive nutrient sources for neonatal piglets, sufficient energy intake during the first days of life is essential for survival [5,6]. To address this challenge, nutritional supplementation strategies, particularly with medium-chain triglycerides (MCTs), have been proposed to provide rapidly absorbable energy that supports early growth and reduces mortality in low-birthweight piglets [7,8].

Medium-chain fatty acids (MCFAs) and their derivatives exert multiple beneficial effects in swine [9]. Among these, medium-chain triglycerides (MCTs) are rapidly absorbed and metabolized, providing readily available energy to neonatal piglets with limited glycogen reserves, supporting thermoregulation, intestinal development, and early growth. In addition to their energetic role, MCTs—particularly those enriched with lauric acid (C12) and its derivatives—exert important antimicrobial, antiviral, and immunomodulatory effects in swine [10,11,12,13]. Studies in swine have demonstrated that low-level supplementation with MCTs can stabilize gut microbiota, support intestinal integrity, and reduce gastrointestinal disturbances across different production stages [9,14]. For example, a 2% dietary blend of caproic (C6:0), caprylic (C8:0), and capric acids (C10:0) in equal ratios inhibited porcine epidemic diarrhea virus (PEDV), improved intestinal morphology, and reduced viral load, thereby enhancing growth performance and mitigating PEDV infectivity [15]. Similarly, there have been reports on the use of calcium laurate (C12-Ca) at approximately 1 g/kg feed, which improved feed efficiency, increased gastric acidity, reduced diarrhea, and attenuated inflammation and oxidative stress, thereby demonstrating beneficial effects at relatively low supplementation levels [16,17]. By contrast, higher levels of lauric acid have been associated with strong antiviral activity, including suppression of PEDV, alongside improvements in feed efficiency and reductions in diarrhea incidence [18]. At even greater concentrations (~60%), lauric acid and its monoglyceride monolaurin have been shown to activate G-protein coupled receptor 84 (GPR84), thereby stimulating cytokine signaling and leukocyte activity, and ultimately enhancing innate immune responses [13]. These findings suggest that low and high doses of lauric acid-based MCT formulations confer distinct but complementary benefits, ranging from gut microbiota stabilization to immune activation, supporting their application as nutraceutical alternatives to antibiotic prophylaxis in swine production.

Phytocannabinoids are a group of lipophilic bioactive compounds derived primarily from hemp (*Cannabis sativa*). Their lipophilic nature underlies their biological activity and contributes to their interaction with cellular membranes and signaling pathways [19]. Functionally, phytocannabinoids exhibit antioxidant and anti-inflammatory effects by inhibiting lipoxygenase activity, stabilizing radical intermediates, and reducing free radical reactivity, thereby mitigating oxidative stress and attenuating inflammation at the cellular level [20,21,22]. In addition, their interactions with cannabinoid receptors modulate immune responses, supporting gut integrity, nutrient absorption, and systemic immunity [23,24]. These components collectively enhance gut integrity, nutrient absorption, and systemic immunity, outcomes that are reflected by increased mean corpuscular hemoglobin concentration, elevated serum total protein and globulin, and reduced eosinophil counts [14]. Due to their fat-soluble properties, oil-based formulations serve as an effective medium to improve the stability and bioavailability of phytocannabinoids [23]. When included in medium-chain triglyceride (MCT) emulsions, Phytocannabinoids may enhance the energetic function of MCTs and their positive impact on the growth and survival of neonatal piglets. Based on this rationale, it was hypothesized that phytocannabinoid-enriched MCT emulsions would improve early growth and immunity, and that the addition of monolaurin would further reduce mortality through antimicrobial effects. These considerations suggest that MCT-based emulsions incorporating phytocannabinoids, with or without additional monolaurin, represent a promising nutritional approach that warrants investigation as an alternative to antibiotics in pig production.

Therefore, this study aimed to evaluate the effects of MCT-based emulsions enriched with phytocannabinoids and monolaurin on growth performance and health indicators, including serum biochemical parameters, pre-weaning mortality, and diarrhea incidence, in suckling piglets.

## 2. Materials and Methods

The current study was carried out at the farrowing and lactation unit of Wilaiporn Farm, Nakhon Sawan Province, Thailand. All experimental procedures, including animal care and management, were reviewed and approved by the Naresuan University Animal Care and Use Committee (ACUC; approval number 67 01 002, 9 July 2024).

### 2.1. Medium-Chain Triglyceride Emulsion Formulations

Three medium-chain triglyceride (MCT) emulsions were used in this study. The baseline formulation of MCT emulsion (MCTE) was developed under a registered petty patent by Naresuan University, Thailand (Application No. 2503000945).To generate derivative formulations, the phytocannabinoid-enriched emulsion (MCTE-P) was prepared by incorporating extracts obtained from 12% hemp leaves into crude palm kernel oil through a controlled 6-h heating process [20,25]. The cannabinoid profile of the extract was 0.168% cannabidiol (CBD), 0.083% cannabidiolic acid (CBDA), and 0.017% tetrahydrocannabinol (THC). The total THC content was well below the 0.2% legal threshold, ensuring the formulation was non-psychoactive and compliant with Thai safety regulations. A second derivative, MCTE-PM, was produced by supplementing MCTE-P with glycerol monolaurate (monolaurin).

All emulsions were manufactured under standardized procedures, and their fatty acid composition, chemical characteristics, quality parameters, and microbiological safety were verified by a certified analytical laboratory (Table 1).

### 2.2. Animals and Experimental Design

**Figure 1:**
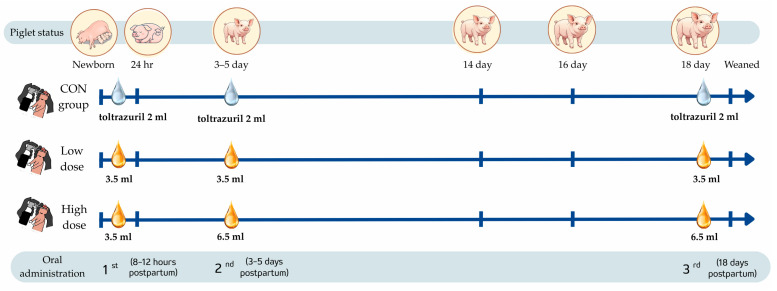
Piglet supplementation schedule showing three critical timepoints: early neonatal (8–12 h), early postnatal (3–5 days), and pre-weaning (16–18 days) periods.

The study was conducted with crossbred piglets (Landrace × Landrace × Duroc), which were born to multiparous sows (parity 3–6). Two trials were conducted to evaluate the efficacy of MCT emulsions as oral supplements in neonatal piglets. Trial 1 was designed to compare the efficacy of a baseline MCT emulsion (MCTE) with that of a phytocannabinoid-supplemented emulsion (MCTE-P), while also determining the most effective oral dosage (low vs. high). Trial 2 employed the optimal formulation and dosage identified in Trial 1 to assess further the additional effect of monolaurin supplementation (MCTE-PM). Both trials compared results with a reference group of piglets receiving standard antibiotic prophylaxis. The experimental timeline, including three critical timepoints, is illustrated in Figure 1.

In Trial 1, seventy-five multiparous sows were randomly allocated into five treatment groups (15 L per group), producing 1063 piglets (average litter size: 14.17 piglets per sow). Oral supplementation was administered at three critical physiological stages: 8–12 h postpartum to address immediate energy needs and hypoglycemia risk, days 3–5 during rapid growth and immune maturation, and days 16–18, preceding weaning, to assess sustained effects on peak growth and pre-weaning health status. Creep feed was withheld for the first 10 days postpartum to isolate the effects of the oral supplement. The trial employed an augmented factorial design (2 × 2 + control), with two factors: (A) emulsion formulation (MCTE vs. MCTE-P) and (B) dosage regimen: low dose (3.5 mL at each of the three administrations) and high dose (3.5 mL at the first, followed by 6.5 mL at the second and third administrations). The control group (CON) received oral toltrazuril at 100 mg per piglet (2 mL of a 5% solution, 50 mg/mL, Toltrazuril 5%) at all three time points [26]. This design enabled assessment of formulation efficacy, dose dependency, and interactions, with the control serving as the reference for standard antibiotic prophylaxis.

In Trial 2, thirty-six multiparous sows (parity 3–6) were randomly allocated into three treatment groups, yielding 509 neonatal piglets. Oral supplementation was administered at three timepoints aligned with Trial 1: the first within 8–12 h after farrowing, the second on day 5, and the third on day 18 postpartum. The trial followed a completely randomized design with three groups: (1) a control group (CON) receiving oral toltrazuril at the same dosage as in Trial 1 (100 mg per piglet, 2 mL of a 5% solution), (2) piglets supplemented with 3.5 mL of MCT emulsion containing phytocannabinoids (MCTE-P), and (3) piglets supplemented with 3.5 mL of the same emulsion further enriched with monolaurin (MCTE-PM).

Both trials included growth, intake, survival, and blood profile measurements, while Trial 2 incorporated behavioral observations to extend the assessment of treatment efficacy.

### 2.3. Productivity and Health Measurements

Productivity and health measurements were collected from piglets born to multiparous sows (parity 3–6) across both trials to ensure consistency in reproductive and lactational performance. Assessments included:

(1) Piglet performance—litter size at birth, number of live-born piglets, and litter size at 5, 14, and 18 days postpartum.
(2) Piglet body weights at 24 h, 5, 14, and 18 days of age, measured with precision digital scales (±0.01 g) by Blavi et al. [27], Chisoro et al. [28], Peltoniemi et al., [29].
(3) Health outcomes—pre-weaning mortality rate, recorded from birth to 24 h and throughout the suckling period, with incidence and causes of death documented.
(4) Nutritional intake—colostrum and milk intake per piglet, calculated using validated equations for suckling piglets adapted by Miguel et al. [30]; Thongkhuy et al. [31].

Colostrum intake (mL/piglet) was calculated as: CI = −106 + 2.26 WG + 200 BW_B_ + 0.111 D − 1414 WG/D + 0.0182 WG/BWB, where WG is piglet weight gain over 24 h (g), BWBs is birth weight (kg), and D is the duration of colostrum suckling (min). The colostrum yield of the sows was defined as the sum of individual colostrum consumption by all piglets in the litter.

Individual milk intake (MI, g/d) was calculated as: MI = (Milk for maintenance) + 1.168 × Gain + 0.00425 × Gain^2^, where milk for maintenance was set at 317 g/day for days 3 to 5.

### 2.4. Hematological and Biochemical Assessments

At 18 days old (8–12 h after the final treatment), piglets weighing 4.5–5.0 kg were randomly chosen from each experimental group for blood sampling. In Trial 1, ten piglets per group were sampled (total *n* = 50), while in Trial 2, eight piglets per group were sampled (total *n* = 24). Blood samples (3–4 mL) were drawn from the cranial vena cava using 18 G × 1.5-inch needles into EDTA tubes and kept at 4–8 °C immediately. Within 30 min, samples were centrifuged at 3000× *g* for 10 min at 4 °C to separate plasma, which was stored at −20 °C within 24 h before lab analysis.

A complete blood count (CBC), including erythrocyte, leukocyte, and platelet parameters, was performed using an ABX Pentra 60 Hematology Analyzer (Horiba, Grabels, France) to assess hematological profiles [32,33]. Plasma protein profiles, comprising total protein, albumin, and globulin concentrations, were quantified using standardized biochemical assays following veterinary clinical pathology guidelines [34]. Total protein concentration was determined using the Biuret method [35], while albumin was measured by an optimized bromocresol green binding assay on a PKL PPC 125 Automatic Chemistry Analyzer (Pokler Italia, Modena, Italy) [36]. Globulin concentration was calculated as the difference between total protein and albumin.

### 2.5. Mortality and Welfare-Related Assessments

Data collection in both trials extended beyond productivity measures to obtain a comprehensive understanding of piglet welfare, including mortality classification, behavioral observations, and physical indicators. Mortality was recorded and categorized to identify underlying causes of death, while additional behavioral and physical assessments were performed in Trial 2 to explore potential factors contributing to survival and early-life health.

#### 2.5.1. Mortality Classification

In both trials, mortality events were systematically documented and classified according to a framework adapted from established protocols in previous studies [16,25,37]. Mortality events were recorded immediately upon discovery. Deaths were categorized into four principal causes based on established clinical and observational criteria:

(1) Milk starvation—characterized by emaciation and milk deprivation, evidenced by frothing at the mouth and severe wasting.
(2) Weak state—referring to piglets that died due to poor vigor and chronic weakness, typically manifested as small body size, visible skeletal structures, or ongoing disease.
(3) Crushing—diagnosed when piglets were found flattened beneath the sow with distinctive purplish swelling at compression sites.
(4) Diarrhea—identified by persistent enteric distress, including yellow staining around the anus, fecal contamination, and malodor.

All cases were recorded immediately upon discovery to ensure accurate documentation. This classification provided important insights into the predominant causes of mortality in neonatal piglets and served as a foundation for extending the investigation to behavioral aspects in Trial 2.

#### 2.5.2. Behavioral and Lesion Assessments

Following observations in Trial 1 that suggested behavioral factors may contribute to mortality risk, additional behavioral assessments were incorporated into Trial 2. For this purpose, eighteen litters containing piglets with low birth weights (1.20–1.50 kg) were selected, and a total of 108 piglets (6 per litter; 36 per treatment group) were included in the behavioral study. Each piglet was individually marked with a 2 × 3-inch neo tape tag placed on the back to allow accurate identification and monitoring. Behavioral data were recorded using overhead video cameras positioned 1.8–2.0 m above the sow pens, and observations were evaluated during the first five days postpartum.

Behavioral evaluations were conducted at two postpartum periods: during the initial 8–12 h after birth and again at 3–5 days postpartum. The assessments were categorized into three domains:

(1) Suckling frequency (SF), defined as the number of approaches each piglet made to the sow’s udder within a two-hour period after treatment [22].
(2) Latency to first suckling (LFS), measured as the time interval (minutes) from release after supplementation until the piglet successfully suckled, providing an index of neonatal vigor and colostrum acquisition [21].
(3) Teat competition and establishment (TOE), quantified by recording the frequency of aggressive interactions or attempts to secure a teat within the same two-hour window [38].

**Figure 2:**
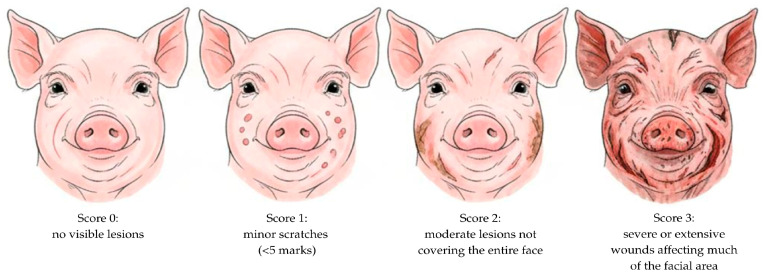
Grading scale for facial lesion scores (0–3) in neonatal piglets, with 0 = no visible lesions, 1 = minor scratches, 2 = moderate lesions, and 3 = severe or extensive wounds.

In addition to behavioral indicators, facial lesion scoring (FLS) was used as a physical measure of social competition. Lesions were evaluated daily between 10:00 and 12:00 h from day 1 to day 5 postpartum. A four-point scoring scale was applied, where 0 = no visible lesions, 1 = minor scratches (<5 marks), 2 = moderate lesions not covering the entire face, and 3 = severe or extensive wounds affecting much of the facial area. Representative scoring criteria are illustrated in Figure 2. The scoring method was adapted from protocols described by Thongkhuy et al. [31] and Zhang et al. [15].

### 2.6. Statistical Analysis

Data analysis was conducted using the General Linear Model (GLM) procedure in SPSS version 26 (IBM Corp., Armonk, NY, USA) to evaluate the effects of emulsion formulations relative to antibiotic control. Data were first tested for normality (Shapiro–Wilk test) and homogeneity of variance (Levene’s test) to confirm the validity of parametric analyses. To account for potential variation arising from differences in initial litter size, litter size at birth was included as a covariate in the GLM for all relevant performance variables, including piglet weight, average daily gain, and mortality rates.

In Trial 1, the augmented factorial design (2 × 2 + control) was examined with two factors: (A) emulsion formulation (MCTE vs. MCTE-P) and (B) dosage level (low vs. high), along with their interaction. The effects of treatment were subsequently analyzed using Tukey’s Honest Significant Difference (HSD) test for pairwise comparisons upon the identification of significant main or interaction effects. Orthogonal contrasts were utilized to evaluate specific hypotheses: (1) control compared to all emulsion treatments and (2) dosage effects within emulsion groups. In Trial 2, the complete randomized design was evaluated using one-way ANOVA, with treatment as the primary effect (Control, MCTE-P, MCTE-PM). All results are presented as means ± standard error of the mean (SEM), with statistical significance defined at *p* ≤ 0.05.

## 3. Results

### 3.1. Trial 1: Effects of MCT Emulsion Formulations and Dosage Levels on Piglet Growth Performance, Colostrum and Milk Intake, and Hematological Parameters

#### 3.1.1. Piglet Performance

In Trial 1, the augmented factorial design evaluated two primary factors: Factor A examined emulsion formulations (MCTE vs. MCTE-P), while Factor B assessed dosage levels (low; L vs. high; H), with a toltrazuril-treated control group serving as the reference standard. The comprehensive assessment included growth performance parameters, colostrum and milk intake measurements, pre-weaning mortality rates, and birth weight-stratified responses to determine optimal treatment protocols (Table 2).

At 24 h postpartum, piglets receiving MCTE-P formulations demonstrated significantly superior weight gain in both L-MCTE-P (0.13 kg) and H-MCTE-P groups (0.13 kg) compared with CON (0.09 kg) and H-MCTE (0.09 kg; *p* = 0.001), indicating enhanced early metabolic adaptation and energy utilization. Colostrum intake reached its maximum in H-MCTE-P (334.63 mL/piglet), significantly higher than CON (287.69 mL/piglet) and L-MCTE (279.93 mL/piglet; *p* = 0.006). Statistical analysis revealed that formulation type exerted a significant main effect (*p* < 0.05), while the absence of formulation × dose interaction (*p* > 0.05) confirmed that these factors operate independently, allowing for precise optimization of both parameters.

At day 14, growth performance reached optimal levels in H-MCTE-P (170.98 g/day) and H-MCTE group (177.06 g/day), both significantly exceeding control performance (120.97 g/day; *p* = 0.010), with corresponding weight gain differentials confirming sustained treatment efficacy (*p* = 0.001). By weaning, growth differences among groups were less pronounced.

#### 3.1.2. Mortality Rate & Causes

Pre-weaning mortality rates were significantly lower in H-MCTE-P (19.69%) and L-MCTE-P (22.65%) compared to CON (29.44%) and MCTE groups (32.69%, 35.82%; *p* = 0.016), with formulation effects showing statistical significance (*p* = 0.001). Mortality attribution analysis indicated significant decreases in deaths due to starvation (5.36% in H-MCTE-P) and diarrhea (3.17% in H-MCTE-P), in contrast to CON and L-MCTE groups, which demonstrated diarrhea-related mortality rates exceeding 8% (*p* < 0.05; Table 2). This indicates that MCTE-P improved early nutrient absorption, litter integrity, and mortality risks in piglets.

#### 3.1.3. Hematological and Biochemical Profiles

The comprehensive hematological analysis in Table 3 revealed significant treatment-induced alterations in erythrocyte indices and leukocyte populations among piglets receiving MCTE-P supplementation, particularly at high dosage levels. Piglets administered H-MCTE-P demonstrated substantially elevated mean corpuscular hemoglobin concentration (MCHC) and red cell distribution width (RDW) compared with control animals (*p* < 0.05), indicating enhanced erythropoietic activity and improved cellular hemoglobin synthesis capacity. The eosinophil percentages demonstrated significant decreases in MCTE-P treated groups (*p* < 0.05), indicating reduced allergic and parasitic inflammatory responses, which are associated with enhanced immune homeostasis and diminished systemic inflammatory response.

Significantly, essential hematological parameters, such as total hemoglobin concentrations, hematocrit values, and total leukocyte counts, remained within physiological range across all treatment groups (*p* > 0.05), thereby affirming the safety profile of the Trial l formulations and suggesting that advantageous effects were achieved without impairing baseline hematopoietic function or cellular carrying capacity.

Furthermore, biochemical profiling revealed significant treatment-related enhancements in protein metabolism and immune competence indicators, with serum total protein (TP) and globulin (GLOB) levels significantly elevated in all EMPL treatment groups compared to controls (*p* < 0.001), achieving peak levels in H-MCTE-P and H-MCTE groups (Table 4). However, there were no significant treatment-related changes in albumin, glucose, and triglyceride concentrations (*p* > 0.05).

### 3.2. Trial 2: Comparative Efficacy of MCTE-P and Monolaurin-Fortified MCT Emulsions (MCTE-PM) on Piglet Performance and Physiological Parameters

#### 3.2.1. Piglet Performance

This trial used the optimal formulation and dosage determined in Trial 1 to further evaluate the additional effect of monolaurin (MCTE-PM). The effects of oral administration of medium-chain triglyceride emulsion containing phytocannabinoids, with or without monolaurin, on the performance of suckling piglets is presented in Table 4.

During the critical 24-h postpartum assessment period, piglets receiving either MCTE-P or MCTE-PM emulsions demonstrated significantly enhanced weight gain (0.13 kg in both treatment groups) compared with control subjects (0.08 kg; *p* = 0.001). The colostrum intake per piglet was significantly higher in both treatment groups, with MCTE-P (328.73 mL) and MCTE-PM (336.73 mL) compared to the control group (276.76 mL; *p* = 0.006). There are no significant differences in piglets at 5 days old; however, the MCTE-P group exhibits the highest average daily gain at 14 days (*p* = 0.031). Finally, during weaning, piglets in the MCTE-P and MCTE-PM groups attained the highest average daily gains of 173.67 and 172.38 g/day, significantly exceeding the control group (107.81 g/day; *p* = 0.043).

#### 3.2.2. Mortality Rate & Causes

The temporal progression of performance benefits was particularly evident in litter size maintenance and pre-weaning mortality reduction, which directly correlates to commercial production efficiency and economic viability. The litter size on day 14 was significantly greater in the MCTE-PM group (11.42 piglets/sow) compared to the control and MCTE-P groups (10.50 and 10.20 piglets/sow, respectively; *p* = 0.031), suggesting improved survival rates and reduced early mortality during the critical pre-weaning period.

This survival advantage was further substantiated by the comprehensive mortality analysis, which revealed that pre-weaning mortality over the complete 18-day lactation period was lowest in MCTE-PM (14.27%), intermediate in MCTE-P (23.63%), and highest in controls (29.40%; *p* = 0.012), representing a remarkable 15.13% reduction in mortality when comparing MCTE-PM to control treatment.

The mortality reduction was most pronounced for deaths attributed to diarrhea, with MCTE-PM demonstrating exceptional protective efficacy (0.86% vs. 10.4% in controls; *p* = 0.001), and starvation-related mortality (4.30% vs. 14.4% in controls; *p* = 0.002), supporting the formulation’s multifaceted protective effects against the most common causes of neonatal piglet losses. The parameters of mortality rate and causes are presented in Table 4.

#### 3.2.3. Piglet Behavior

**Figure 3:**
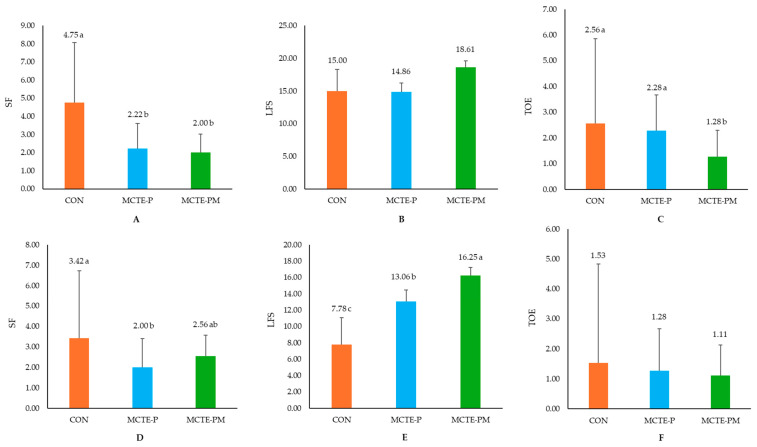
Behavioral responses of piglets following oral administration of experimental emulsions: (**A**) suckling frequency (SF), (**B**) latency to first suckling (LFS), and (**C**) teat order establishment (TOE) during the initial 8–12 h postpartum; and following the second administration of experimental emulsions: (**D**) SF, (**E**) LFS, and (**F**) TOE at 3–5 days postpartum. Mean followed by different superscript letters (^a–c^) from each other indicate significant differences (*p* < 0.05).

Behavioral analysis indicated that piglets in the control (CON) group showed the highest mean suckling frequency (SF), latency to first suckling (LFS), and teat order establishment (TOE) compared to both MCTE-P and MCTE-PM groups during both the initial 8–12 h and at 3–5 days postpartum (*p* < 0.05). Suckling frequency was significantly greater in the CON group than in MCTE-P and MCTE-PM (*p* < 0.05; Figure 3A,D). The MCTE-PM group exhibited the highest mean LFS during the second feeding (16.25 min) compared to MCTE-P (13.06 min) and control group (7.78 min; *p* > 0.05; Figure 3E). Aggressive teat order behaviors (TOE) were most frequent in the CON group in the first 8–12 h but significantly reduced in the MCTE-PM group (*p* < 0.05), indicating less inter-piglet conflict (Figure 3C).

**Figure 4:**
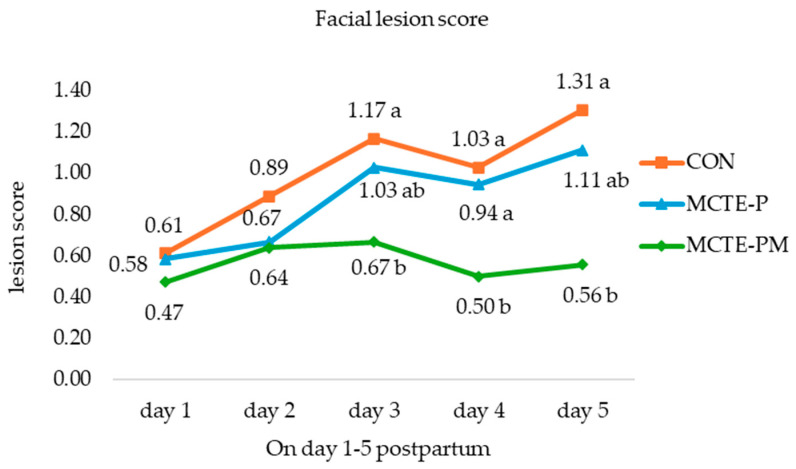
Facial lesion scores of piglets from day 1 to day 5 postpartum across treatment groups. Mean followed by different superscript letters (^a,b^) from each day indicate significant differences (*p* < 0.05).

Daily facial lesion scores (FLS) of piglets from day 1 to day 5 postpartum are presented in Figure 4. Lesion scores increased progressively in both the control (CON) and MCTE-P groups, reaching their highest values on day 5 of the observation period. FLS showed a progressive increase in both the control (CON) and MCTE-P groups, peaking on day 5 with scores of 1.31 and 1.11, respectively. In contrast, piglets in the MCTE-PM group consistently exhibited the lowest FLS across the experimental period, with a final score of 0.56 on day 5. Statistically significant differences (*p* < 0.05) were observed between CON and MCTE-PM on days 3, 4, and 5. The findings demonstrate that MCTE-PM supplementation significantly alleviated facial trauma and diminished teat-related injuries in neonatal piglets by decreasing inter-piglet aggression.

#### 3.2.4. Hematological and Biochemical Profiles

Hematological parameters of piglets are summarized in Table 5. Red blood cell (RBC) counts were significantly lower in the MCTE-PM group (5.98 × 10^6^/µL) compared with both CON and MCTE-P (*p* = 0.003). Nevertheless, all values remained within the physiological reference range for weaned piglets (4.98–8.29 × 10^6^/µL). Mean corpuscular volume (MCV) was significantly higher in MCTE-P (57.13 fL) and MCTE-PM (60.50 fL) relative to CON (52.50 fL; *p* = 0.008), reflecting the presence of larger, more mature red cells suggestive of improved iron utilization and enhanced hemoglobin synthesis. In contrast, mean corpuscular hemoglobin concentration (MCHC) was significantly lower in both supplemented groups (30.0–30.5 g/dL) compared with CON (33.0 g/dL; *p* < 0.001). These values were consistent with reported ranges for healthy suckling piglets, suggesting an optimal distribution of hemoglobin without pathological concentration. Red cell distribution width (RDW) was also elevated in both MCTE-P and MCTE-PM groups (*p* = 0.009).

Regarding leukocyte profiles, a marked reduction in eosinophil percentage was observed in MCTE-PM (0.25%) compared with MCTE-P (2.00%) and CON (3.75%; *p* = 0.002), indicating improved immune modulation.

Biochemical analyses demonstrated that both MCTE-P and MCTE-PM significantly elevated total protein concentrations (5.16, 5.13 g/dL) and globulin levels (1.51, 1.76 g/dL) compared to the control group (4.00 and 0.61 g/dL, respectively; *p* < 0.001), while albumin concentrations remained consistent across groups (*p* > 0.05).

## 4. Discussion

This study evaluated the effects of medium-chain triglyceride (MCT) emulsions enriched with phytocannabinoids, with or without monolaurin, on neonatal piglet growth, health, and survival. Two consecutive trials were conducted to assess performance relative to conventional antibiotic prophylaxis.

In Trial 1, the efficacy of MCT emulsions was evaluated by comparing a baseline formulation (MCTE) and a phytocannabinoid-enriched formulation (MCTE-P) with a standard toltrazuril group. As shown in Table 2, piglets receiving either MCT emulsion performed comparably or better than the antibiotic control. This highlights the metabolic advantage of MCTs, which provide rapid, efficient energy through direct absorption into the portal vein, supporting thermoregulation, gut maturation, and immune function in neonatal piglets [7,8]. In addition, lauric acid, a major constituent of these emulsions, is known to exert broad-spectrum antimicrobial activity, which can support gut health [39]. This is complemented by its antiviral effects against pathogens such as porcine epidemic diarrhea virus (PEDV) [13]. These properties support neonatal health and survival, and piglets receiving phytocannabinoid-enriched emulsions (MCTE-P) showed better outcomes than controls (Table 2). Both low-dose (L-MCTE-P) and high-dose (H-MCTE-P) groups showed significantly greater body weight gain within the first 24 h postpartum, highlighting improved early nutrient utilization and vigor. Notably, colostrum intake was highest in the H-MCTE-P group, which likely enhanced passive immunoglobulin transfer and contributed to sustained growth benefits [14,15].

Mortality analyses showed that overall pre-weaning mortality did not differ significantly among most groups; however, the H-MCTE-P group recorded a significantly lower total mortality rate (19.69%) compared with the control (29.44%). Importantly, cause-specific analyses revealed that piglets receiving either low- or high-dose MCTE-P had markedly fewer deaths from diarrhea and milk starvation relative to controls. These reductions can be explained by the complementary actions of lauric acid, a principal component of MCT emulsions, and phytocannabinoids. Lauric acid exerts direct antimicrobial effects by disrupting pathogen membranes and strengthening gut barrier integrity [10,15,40] thereby limiting enteric infections and improving nutrient utilization. In parallel, phytocannabinoids modulate systemic inflammation and enhance immune responsiveness through endocannabinoid pathways [23,41], helping piglets resist infection and tolerate early-life stressors. Together, these mechanisms may have contributed to reducing the susceptibility of MCTE-P piglets to diarrhea- and starvation-related mortality. By contrast, crushing mortality was significantly higher in the H-MCTE-P group than in controls, a finding possibly linked to increased activity and competition for teat access; this phenomenon is further addressed in the behavioral analyses of Trial 2.

Hematological and biochemical analyses further supported the functional advantages of MCTE-P supplementation. Both low- and high-dose MCTE-P piglets exhibited reduced mean corpuscular hemoglobin concentration (MCHC) and elevated red cell distribution width (RDW), reflecting active erythropoiesis and improved erythrocyte turnover consistent with enhanced growth performance. In addition, serum total protein and globulin concentrations were significantly higher in MCTE-P groups compared with controls (Table 3), indicating improved passive immunity through more efficient colostrum absorption [35,42]. Eosinophil percentages were also markedly reduced in both MCTE-P groups, particularly in the high-dose group, suggesting diminished systemic inflammation and better immune regulation. These results align with the antimicrobial and gut-stabilizing effects of lauric acid [7,15] and the anti-inflammatory properties of phytocannabinoids [35,41], which together contributed to improved immune competence in neonatal piglets. Accordingly, these findings show that both low- and high-dose MCTE-P benefited piglets compared with controls, particularly by enhancing immune responses and reducing diarrhea-related mortality. However, as overall mortality reduction was similar between regimens and the high-dose posed practical challenges, the low-dose MCTE-P was chosen for further evaluation in Trial 2.

Building on this rationale, Trial 2 was designed to confirm the efficacy of the low-dose MCTE-P regimen alongside a monolaurin-fortified formulation (MCTE-PM). The addition of monolaurin, a lauric acid ester with established antimicrobial activity, was intended to enhance efficacy while maintaining practical feasibility for routine administration. In terms of growth performance as shown in Table 4, both MCTE-P and MCTE-PM improved average daily gain (ADG) at weaning relative to controls, confirming the benefits observed in Trial 1. These gains were accompanied by trends of greater early weight gain and enhanced colostrum intake, which likely improved passive immunoglobulin transfer and supported sustained growth [43]. Such outcomes reflect more efficient nutrient utilization and metabolic optimization during the most vulnerable period of piglet development [41,44]. The addition of monolaurin significantly impacted survival outcomes. MCTE-PM achieved the lowest pre-weaning mortality (14.27%) compared with both the control group (29.40%) and MCTE-P (23.63%) (Table 4). Diarrhea-related deaths, which represented a major cause of early mortality, were reduced dramatically from 10.4% in controls to only 0.86% in the MCTE-PM group (*p* < 0.001). These results support the synergistic activity of lauric acid and monolaurin in limiting pathogen proliferation and preserving gut barrier function, alongside the immunomodulatory role of phytocannabinoids in reducing inflammatory burden [28,45]. As in Trial 1, however, crushing mortality remained higher in emulsion-treated groups than in controls, suggesting that increased vigor and competitiveness may have offset some of the survival gains. To address this possibility, behavioral outcomes were specifically assessed in Trial 2.

Behavioral assessments provided further insight into the higher crushing mortality observed in emulsion-treated piglets. Among 108 low-birth-weight piglets, those supplemented with MCTE-PM displayed notably calmer behavioral patterns (Figure 3). Latency to first suckling was prolonged relative to controls, yet teat competition and aggression were markedly reduced, as evidenced by lower rates of teat disputes and significantly reduced facial lesion scores (0.56 on day 5) (Figure 4). These findings indicate that MCTE-PM facilitated more orderly access to the udder and diminished social stress, which may mitigate some of the risks associated with increased vigor and competitiveness in supplemented piglets [38,46]. From a production perspective, the reduction in aggressive suckling behavior is particularly advantageous in systems where teeth clipping is not practiced, as it minimizes injury and improves welfare while supporting efficient colostrum intake. Thus, while increased energy supply may elevate competition in some contexts, the addition of monolaurin appeared to balance this effect by promoting calmer suckling behavior and reducing harmful interactions [7].

Hematological and biochemical profiles in Trial 2 further supported the survival advantages observed with emulsion supplementation (Table 5). Eosinophil percentages were lowest in the MCTE-PM group (*p* < 0.05), indicating reduced systemic inflammatory activation and improved disease resilience [47]. At the same time, serum total protein and globulin concentrations remained elevated in both MCTE-P and MCTE-PM piglets compared with controls (*p* < 0.001), consistent with enhanced passive immunity acquisition through improved colostrum intake. These effects align with the immunomodulatory actions of phytocannabinoids and the antimicrobial properties of monolaurin and lauric acid, reinforcing the reductions in diarrhea-related mortality seen in treated groups [42,46,48,49].

Taken together, these results identify MCTE-PM as the most efficacious formulation, combining the rapid energy supply of MCTs, the anti-inflammatory and immunomodulatory actions of phytocannabinoids, and the antimicrobial activity of monolaurin. This synergistic approach provided measurable improvements in growth, survival, immunity, and behavior, highlighting its potential as a nutraceutical alternative to conventional antibiotic prophylaxis in sustainable swine production systems [18,50,51,52,53]. Nonetheless, the trial was limited to the pre-weaning period (18 days), excluded microbiota analyses, and may not fully predict commercial-scale outcomes. Future work should focus on gut microbiome analysis and improving formulation stability and spray-drying methods to enable large-scale application and practical adoption in commercial farms.

## 5. Conclusions

This study demonstrated that medium-chain triglyceride emulsions provided overall performance comparable to antibiotic prophylaxis in neonatal piglets. Supplementation with phytocannabinoids (MCTE-P) further reduced diarrhea-related mortality to levels significantly lower than the control group. Moreover, the monolaurin-fortified formulation (MCTE-PM) consistently showed the greatest efficacy, with significantly improved average daily gain at weaning, enhanced hematological and immunological profiles, and lower overall mortality rate, primarily resulting from a marked reduction in diarrhea-related deaths. In addition, piglets receiving MCTE-PM displayed reduced aggressive suckling behaviors, including lower teat competition and facial lesion scores. These findings suggested that MCT-based emulsions, particularly MCTE-PM, are promising nutraceutical alternatives to antibiotics for enhancing piglet health, welfare, and survival in commercial swine production.

## 6. Patents

Thailand petty patent application (No. 2503000945) was filed on 14 March 2025 for the invention related to this research. The title of the invention is “Medium-chain fatty acid oil powder formula containing Phytocannabinoid extract and glycerol monolaurate and production method” The inventors are Wandee Tartrakoon, Rangsun Charoensook, and Adisak Kongkeaw. All rights to this invention are owned by Naresuan University.

## Figures and Tables

**Table 1:** Fatty acid and chemical composition of medium-chain triglyceride emulsions (MCTE, MCTE-P, and MCTE-PM) used in Trials 1 and 2.

Item ^1^	MCTE	MCTE-P	MCTE-PM
Fatty acid composition (g/100 g)			
Caproic acid (C6:0)	0.09	0.09	0.09
Caprylic acid (C8:0)	1.38	1.26	1.12
Capric acid (C10:0)	1.37	1.25	1.21
Lauric acid (C12:0)	33.94	41.42	41.23
Medium-chain fatty acids (MCFA)	36.80	44.04	43.67
Myristic acid (C14:0)	5.97	5.93	5.48
Palmitic acid (C16:0)	7.62	9.08	8.22
Stearic acid (C18:0)	1.43	1.45	1.42
Arachidic acid (C20:0)	0.16	0.19	0.15
Behenic acid (C22:0)	0.07	0.09	0.07
Lignoceric acid (C24:0)	0.07	0.08	0.06
Saturated Fatty acid (g/100 g)	52.10	60.84	59.05
Palmitoleic acid (C16:1n7)	0.05	0.05	0.05
Trans-9-Elaidic acid (C18:1n9-t)	0.03	0.06	0.05
cis-9-0leic acid (C18:1n9-c)	13.21	14.92	13.81
cis-11-Eicosenoic acid(C20:1n11-c)	0.13	0.15	0.12
Nervonic acid (C24:1n9)	0.03	0.05	0.04
Monounsaturated fatty acid (g/100 g)	13.46	15.12	14.08
cis-9,12-Linoleic acid (C18:2n6)	7.31	7.71	7.04
gamma-Linoleic acid (C18:3n6)	0.03	0.04	0.03
alpha-Linoleic acid (C18:3n3)	0.40	0.59	0.48
cis-11,14-Eicosadienoic acid (C20:2)	0.06	0.03	0.03
Arachidonic acid (C20:4n6)	0.01	0.01	0.01
Polyunsaturated Fatty acid (g/100 g)	7.66	7.59	7.38
Unsaturated fat (g/100 g)	21.32	22.42	21.64
Omega 3 (mg/100 g)	442.00	498.42	482.64
Omega 6 (mg/100 g)	7358.63	7761.14	7478.79
Omaga 9 (mg/100 g)	13,242.06	14,270.29	12,841.89
Chemical composition			
Ash	0.12	0.12	0.10
Calories from Fat (kcal/100 g)	661.59	788.94	748.78
Carbohydrates (g/100 g)	<0.01	<0.01	<0.01
Fat (g/100 g)	73.51	87.65	82.42
Water Content (%)	15.23	15.42	15.32
Iodine Value (%)	47.85	41.89	42.30
Peroxide Value (mEq Peroxide/kg)	3.7	4.73	5.23

^1^ MCTE = baseline MCT emulsion; MCTE-P = MCTE supplemented with phytocannabinoids; MCTE-PM = MCTE-P further supplemented with monolaurin.

**Table 2:** The effects of oral administration of medium-chain triglyceride emulsions containing lauric acid with or without Phytocannabinoids, and the dose levels, on the performance of suckling piglets (Trial 1).

Parameters	CON	MCTE		MCTE-P		SEM	*p*-Value from Orthogonal Contrast				
		L-MCTE	H-MCTE	L-MCTE-P	H-MCTE-P		CON vs. Emulsion	CON vs. MCTE vs. MCTE-P	A	B	A × B
Number of sows	15	15	15	15	15						
Newborn piglets											
Number of piglets	229	197	220	208	209						
Litter size (piglet/sow)	15.27	13.13	14.67	13.87	13.93	0.31	0.074	0.224	0.353	0.244	0.285
Birth weight (kg)	1.37	1.31	1.31	1.25	1.28	0.02	0.108	0.465	0.320	0.728	0.706
Live born piglets (24 h)											
Number of piglets	202	188	199	191	196						
Weight (kg)	1.49	1.43	1.42	1.36	1.42	0.02	0.105	0.419	0.414	0.634	0.422
Weight gain (kg)	0.09 ^b^	0.11 ^ab^	0.09 ^b^	0.13 ^a^	0.13 ^a^	0.01	0.020	0.001	0.001	0.303	0.139
Colostrum intake (mL/piglet)	287.69 ^b^	310.51 ^ab^	279.93 ^b^	325.04 ^ab^	334.63 ^a^	5.61	0.079	0.006	0.003	0.355	0.080
Piglets at 5 days old											
Number of piglets	183	150	160	171	167						
Weight (kg)	2.13	1.87	1.91	1.74	1.96	0.05	0.038	0.173	0.706	0.203	0.415
ADG (g/day/piglet)	156.29	100.72	132.87	104.90	117.07	8.55	0.046	0.228	0.686	0.127	0.488
Milk intakes (mL/piglet)	567.49	501.84	586.41	530.86	545.59	17.63	0.554	0.611	0.883	0.218	0.385
Piglets at 14 days old											
Number of piglets	167	146	143	165	165						
Weight (kg)	3.21	3.57	3.44	3.33	3.52	0.08	0.197	0.604	0.647	0.855	0.364
ADG (g/day/piglet)	120.97 ^b^	164.24 ^ab^	155.47 ^ab^	177.06 ^a^	170.98 ^a^	7.19	0.010	0.106	0.366	0.635	0.931
Weaned pigs (at 18 days old)											
Number of piglets	143	127	127	148	157						
Litter size (piglet/sow)	9.50	8.43	8.52	9.88	10.49	0.18	0.446	0.754	0.285	0.680	0.804
Weight (kg)	4.27	4.67	4.20	4.29	4.21	0.11	0.783	0.664	0.424	0.253	0.402
ADG (g/day/piglet)	163.16	170.67	164.91	171.16	179.93	6.34	0.595	0.569	0.363	0.172	0.589
Mortality rate & causes											
Mortality rate (%)	29.44 ^ab^	32.69 ^ab^	35.82 ^a^	22.65 ^bc^	19.69 ^c^	1.68	0.744	0.016	0.001	0.787	0.238
Mortality causes											
Milk starvation (%)	15.40 ^a^	10.95 ^ab^	10.20 ^ab^	6.14 ^ab^	5.36 ^b^	0.96	0.037	0.073	0.050	0.371	0.606
Weak state (%)	1.33 ^b^	6.07 ^a^	6.30 ^a^	3.35 ^ab^	2.40 ^b^	0.55	0.002	0.009	0.010	0.772	0.636
Crushing (%)	2.47 ^c^	7.34 ^b^	13.31 ^a^	10.20 ^ab^	8.76 ^b^	0.77	0.001	0.001	0.599	0.162	0.024
Diarrhea (%)	10.24 ^a^	8.33 ^a^	6.01 ^ab^	2.96 ^b^	3.17 ^b^	0.74	0.015	0.008	0.002	0.417	0.331

Note: Different superscript letters (^a–c^) within the same row indicate significant differences (*p* < 0.05). SEM, standard error of the mean Factor A, emulsion formulation (MCTE vs. MCTE-P); Factor B, dosage level (low vs. high); Factor A × B, interaction between formulation and dosage level; CON, antibiotic control. MCTE, baseline MCT emulsion; MCTE-P, MCTE supplemented with phytocannabinoids; L-, low-dose regimen (3.5 mL at all timepoints); H-, high-dose regimen (3.5 mL initial, then 6.5 mL at subsequent administrations).

**Table 3:** The effects of oral administration of medium-chain triglyceride emulsions with or without phytocannabinoids, and dosage levels, on hematological parameters and serum protein profiles of pre-weaning piglets (Trial 1).

Parameters	CON	MCTE		MCTE-P		SEM	*p*-Value from Orthogonal Contrast				
		L-MCTE	H-MCTE	L-MCTE-P	H- MCTE-P		CON vs. Emulsion	CON vs. MCTE vs. MCTE-P	A	B	A × B
Number of piglets	10	10	10	10	10						
Complete Blood Count (CBC)											
WBC (cell × 10^4^/ mm^3^)	1.28	1.16	1.37	1.14	1.31	0.057	0.830	0.680	0.760	0.163	0.886
RBC (cell × 10^6^/mm^3^)	6.69	6.14	6.24	6.67	6.45	0.087	0.148	0.156	0.053	0.747	0.399
Hemoglobin (g/dL)	11.75	11.07	10.87	11.47	11.36	0.22	0.304	0.742	0.367	0.752	0.927
Hct (%)	36.20	34.00	34.00	38.60	37.70	0.67	0.941	0.089	0.005	0.746	0.746
Platelet count (cell × 10^4^/mm^3^)	58.43	62.21	62.59	59.95	47.72	2.58	0.962	0.356	0.163	0.331	0.301
MCV (fL)	53.90	55.00	54.10	57.40	58.20	0.67	0.179	0.142	0.020	0.970	0.530
MCH (pg)	17.60	18.00	17.40	17.30	17.70	0.22	1.000	0.874	0.692	0.843	0.326
MCHC (g/dL)	32.70 ^a^	32.70 ^a^	32.10 ^a^	29.90 ^b^	30.10 ^b^	0.22	0.005	<0.001	<0.001	0.468	0.151
RDW (%)	17.05 ^b^	17.58 ^b^	17.15 ^b^	19.77 ^a^	21.01 ^a^	0.34	0.029	<0.001	<0.001	0.509	0.178
Neutrophil (%)	38.00	30.50	32.20	28.80	29.80	1.85	0.098	0.557	0.608	0.735	0.930
Lymphocyte (%)	51.40	61.50	58.70	61.90	62.80	1.91	0.039	0.323	0.580	0.815	0.649
Monocyte (%)	6.80	6.00	7.00	7.60	6.50	0.29	0.973	0.512	0.401	0.939	0.114
Eosinophil (%)	3.80 ^a^	2.00 ^ab^	2.10 ^ab^	1.70 ^b^	0.90 ^b^	0.25	<0.001	0.004	0.142	0.488	0.374
Biochemical tests											
Total protein (g/dL)	4.12 ^c^	4.80 ^b^	4.28 ^c^	5.40 ^a^	5.29 ^a^	0.10	<0.001	<0.001	<0.001	0.053	0.200
Albumin (g/dL)	3.33	3.62	3.60	3.69	3.65	0.05	0.011	0.154	0.579	0.781	0.926
Globulin (g/dL)	0.79 ^b^	1.18 ^bc^	0.68 ^b^	1.71 ^a^	1.64 ^ab^	0.09	<0.001	<0.001	<0.001	0.043	0.122

Note: Different superscript letters (^a–c^) within the same row indicate significant differences (*p* < 0.05). SEM, standard error of the mean; Factor A, emulsion formulation (MCTE vs. MCTE-P); Factor B, dosage level (low vs. high); Factor A × B, interaction between formulation and dosage level; CON, antibiotic control; MCTE, baseline MCT emulsion; MCTE-P, MCTE supplemented with phytocannabinoids; L-, low-dose regimen (3.5 mL at all timepoints); H-, high-dose regimen (3.5 mL initial, then 6.5 mL at subsequent administrations). Hematological indices: MCV, mean corpuscular volume (fL); MCH, mean corpuscular hemoglobin (pg); MCHC, mean corpuscular hemoglobin concentration (g/dL); RDW, red cell distribution width (%).

**Table 4:** The effects of medium-chain triglyceride emulsion containing phytocannabinoids, with or without monolaurin, on the performance of suckling piglets (Trial 2).

Parameters	CON	MCTE-P	MCTE-PM	SEM	*p*-Value
Number of sows	12	12	12		
Newborn piglets					
Number of piglets	183	158	168		
Litter size (piglet/sow)	15.25	13.17	14.00	0.45	0.159
Birth weight (kg)	1.35	1.26	1.34	0.03	0.459
Live born piglets (24 h)					
Number of piglets	163	149	160		
Litter size (piglet/sow)	13.61	12.44	13.32	0.46	0.613
Weight (kg)	1.47	1.38	1.47	0.03	0.377
Weight gain (kg)	0.08 ^b^	0.13 ^a^	0.13 ^a^	0.01	0.001
Colostrum intake (mL/piglet)	276.76 ^b^	328.73 ^a^	336.73 ^a^	8.65	0.006
Piglets at 5 days old					
Number of piglets	133	124	144		
Litter size (pigs/sow)	11.08	10.33	12.00	0.25	0.652
Weight (kg)	1.86	1.85	1.90	0.12	0.158
ADG (g/day/piglet)	100.89	108.47	109.00	12.35	0.113
Milk intakes (mL/piglet)	523.14	527.05	514.97	523.14	0.279
Piglets at 14 days old					
Number of piglets	126	122	137		
Litter size (piglet/sow)	10.50 ^b^	10.20 ^b^	11.42 ^a^	0.29	0.031
Weight (kg)	3.13	3.37	3.53	0.12	0.425
ADG (g/day/piglet)	107.81 ^c^	170.58 ^a^	155.57 ^b^	11.62	0.043
Weaned pigs (at 18 days old)					
Number of piglets	115	114	137		
Litter size (piglet/sow)	9.60 ^b^	9.50 ^b^	11.42 ^a^	0.50	0.052
Weight (kg)	4.05	4.36	4.44	0.17	0.623
ADG (g/day/piglet)	151.88 ^b^	173.67 ^a^	172.38 ^a^	9.18	0.046
Mortality rate & causes					
Mortality rate (%)	29.40 ^a^	23.63 ^ab^	14.27 ^b^	2.36	0.012
Mortality causes					
Milk starvation (%)	14.40 ^a^	9.23 ^ab^	4.30 ^b^	1.51	0.002
Weak state (%)	1.68	3.40	1.78	0.57	0.304
Crushing (%)	2.92 ^b^	9.30 ^a^	7.33 ^a^	1.02	0.022
Diarrhea (%)	10.40 ^a^	1.70 ^b^	0.86 ^b^	1.11	0.001

Note: Different superscript letters (^a–c^) within the same row indicate significant differences (*p* < 0.05). SEM, standard error of the mean; CON, antibiotic control; MCTE-P, MCTE supplemented with phytocannabinoids; MCTE-PM, MCTE-P further supplemented with monolaurin.

**Table 5:** Effects of low-dose medium-chain triglyceride emulsions containing phytocannabinoids, with or without monolaurin, on hematological parameters and serum protein profiles of pre-weaning piglets (Trial 2).

Parameters	CON	MCTE-P	MCTE-PM	SEM	*p*-Value
Number of piglets	8	8	8		
Complete Blood Count (CBC)					
WBC (cell × 10^4^/ mm^3^)	1.15	1.24	1.03	0.06	0.303
RBC (cell ×10^6^/mm^3^)	6.82 ^a^	6.80 ^a^	5.98 ^b^	0.12	0.003
Hemoglobin (g/dL)	11.83	11.66	11.06	0.28	0.536
Hct (%)	36.00	39.13	36.25	0.88	0.284
Platelet count (cell × 10^4^/mm^3^)	60.65	55.88	44.89	3.49	0.170
MCV (fL)	52.50 ^b^	57.13 ^a^	60.50 ^a^	1.12	0.008
MCH (pg)	17.38	17.25	18.63	0.31	0.131
MCHC (g/dL)	33.00 ^a^	30.00 ^b^	30.50 ^b^	0.33	<0.001
RDW (%)	16.54 ^b^	19.65 ^a^	20.13 ^a^	0.55	0.009
Neutrophil (%)	35.75	30.88	35.75	2.69	0.712
Lymphocyte (%)	54.13	59.50	57.88	2.83	0.746
Monocyte (%)	6.38	7.63	6.13	0.40	0.265
Eosinophil (%)	3.75 ^a^	2.00 ^b^	0.25 ^c^	0.44	0.002
Biochemical tests					
Total protein (g/dL)	4.00 ^b^	5.16 ^a^	5.13 ^a^	0.15	<0.001
Albumin (g/dL)	3.39	3.65	3.36	0.07	0.155
Globulin (g/dL)	0.61 ^b^	1.51 ^a^	1.76 ^a^	0.14	<0.001

Note: Different superscript letters (^a–c^) within the same row indicate significant differences (*p* < 0.05). SEM, standard error of the mean; CON, control group; MCTE-P, medium-chain triglyceride emulsion supplemented with phytocannabinoids; MCTE-PM, MCTE-P further supplemented with monolaurin; MCV, mean corpuscular volume (femtoliters, fL); MCH, mean corpuscular hemoglobin (picograms, pg); MCHC, mean corpuscular hemoglobin concentration (grams per deciliter, g/dL); RDW, red cell distribution width (%).

## Data Availability

The original contributions presented in the study are included in the article, further inquiries can be directed to the corresponding author.

## Abbreviations

MCTE	Medium-Chain Triglyceride Emulsion
L-MCTE	Low Dose Medium-Chain Triglyceride Emulsion
H-MCTE	High Dose Medium-Chain Triglyceride Emulsion
MCTE-P	Medium-Chain Triglyceride Emulsion Supplemented with Phytocannabinoids
L-MCTE-P	Low Dose Medium-Chain Triglyceride Emulsion with Phytocannabinoids
H-MCTE-P	High Dose Medium-Chain Triglyceride Emulsion with Phytocannabinoids
MCTE-PM	Medium-Chain Triglyceride Emulsion with Phytocannabinoids and Monolaurin
MCFA	Medium-Chain Fatty Acids
MCT	Medium-chain triglycerides
PKO	Palm Kernel Oil
MCHC	Mean corpuscular hemoglobin concentration
